# Factors associated with future fertility intentions among Korean women of childbearing age in Seoul: a cross-sectional study

**DOI:** 10.4069/whn.2024.12.06.1

**Published:** 2024-12-30

**Authors:** Thi Thanh Lan Nguyen, Van Cuong Nguyen

**Affiliations:** 1 Faculty of Korean Language, Phenikaa University, Hanoi City, Vietnam; 2 Department of Otolaryngology-Head and Neck Surgery, Hanyang University College of Medicine, Seoul, Korea

**Keywords:** Childbearing, Fertility, Marriage, Sex education, Sociodemographic factors

## Abstract

**Purpose:**

South Korea currently has the lowest fertility rate among Organization for Economic Co-operation and Development countries, with a total fertility rate of less than one child per woman. This study explored the factors influencing future fertility intentions (FFI) among Korean women of childbearing age.

**Methods:**

Using a cross-sectional design, we analyzed data from the 2022 Survey on Low Birth Policy Demand in Seoul, conducted by the Seoul Women and Family Foundation. This study focused on women of childbearing age. Bivariate analyses and a multivariate logistic regression model were employed to investigate the FFI rate and its associated factors.

**Results:**

The observed overall FFI rate among Korean women of childbearing age was 25.6%, with rates of 27.9% among unmarried women, and 22.0% among married women. Factors associated with FFI included education level, employment status, weekly working hours, number of children, age at first sexual education, monthly income, and perspectives on marriage, childbearing, and gender inequality. High FFI rates were noted among self-employed individuals (33.3%); those who received sexual education in adulthood (48.6%), those who believed in having children after marriage (44.0%), those who thought children strengthen relationships (32.5%), and those who perceived gender equality in family or workplace culture (31.7%).

**Conclusion:**

This study assessed the FFI rate among Korean women of childbearing age, identifying the sociodemographic characteristics and perspectives on marriage, childbearing, and gender inequality that influence it. The findings provide valuable insights for policymakers and nurses to engage with women and increase birth rates in Seoul and throughout South Korea.

## Introduction

The global fertility rate has seen a significant decline over the past five decades, falling from 4.8 children per woman in 1970 to 2.3 in 2022, according to a World Bank report [1]. This data also highlights a substantial reduction in the total fertility rate (TFR) among developed nations. For example, between 1970 and 2022, Canada’s TFR decreased from 2.3 to 1.3, Italy’s from 2.4 to 1.2, Australia’s from 2.9 to 1.6, and Singapore’s from 3.1 to 1.0 [1]. East Asian countries have seen even steeper declines in TFR, surpassing those in Western countries [2]. In China, the TFR dramatically fell from 6.1 in 1970 to 1.2 in 2022, and in Japan, it dropped from 2.1 to 1.3 over the same period [1]. South Korea has also witnessed a significant reduction, with Statistics Korea and the World Bank noting a decrease from 4.5 in 1970 to 1.3 in 2004, further declining to 0.98 in 2018 and 0.8 in 2022, with projections indicating it could drop to 0.68 by 2024 [1,3]. South Korea now records the lowest TFR among Organization for Economic Co-operation and Development countries, with a birth rate below one child per woman.

A low TFR may have far-reaching consequences, such as straining the domestic labor market, exacerbating the challenges associated with an aging population, and increasing the healthcare burden [4]. Furthermore, a persistently low TFR can adversely affect public finances, living standards, and social welfare, while also placing pressure on the government to develop and implement effective policies [5].

Recent studies in Korea have focused on identifying the TFR and the primary factors contributing to its decline [6-9]. Among the discussed reasons are rapid economic growth and changes in labor market trends, which have resulted in an increased number of women joining the workforce, despite persistent gender inequality [6]. While government policies have attempted to promote childbearing, the escalating costs of childcare and education have discouraged many women from having children [6,7]. Additionally, sociodemographic factors, competitive job markets, and rising housing prices in urban areas have placed considerable pressure on individuals [8].

Unlike TFR, future fertility intentions (FFI) serve as an early indicator of potential fertility rates as a reflection of individuals’ desires, intentions, attitudes, and behaviors related to having children [10]. Although FFI may not guarantee future childbearing, it is nevertheless a key predictor of TFR [10]. The FFI rate can be influenced by a variety of factors at both the micro and macro levels, including individual characteristics, subconscious personal motivations, and broader socio-cultural-economic conditions [10]. Numerous studies have examined the FFI rate and its associated factors across various countries, including Hong Kong [5], China [11,12], Australia [13], and several European nations [14-16]. However, most of these studies have primarily focused on sociodemographic factors, housing wealth, and childbirth anxiety, often neglecting perspectives on marriage and childbearing.

The number of studies on FFI among Korean women remains limited. One study focused on newlyweds in South Korea and identified various factors influencing FFI, including demographics, socioeconomics, housing situation, residential satisfaction, and housing expectations [17]. However, its findings have limited generalizability as it excluded women of childbearing age who were unmarried or married but childless and did not explore their perspectives on marriage and childbearing. Another recent study broadened the research sample to include all women of childbearing age but primarily concentrated on the impact of working hours on FFI, neglecting to explore women’s perspectives on marriage and childbearing [18]. These gaps in existing research underscore the need for a comprehensive study that addresses these limitations.

In this study, we determined the FFI rate and evaluated the influence of sociodemographic factors, as well as attitudes toward marriage, childbearing, and gender inequality, on FFI among Korean women of childbearing age. To the best of our knowledge, this issue has not been comprehensively addressed in previous research within Korea. Therefore, our findings could inform policy development aimed at boosting the country’s birth rate, which is a major concern for both the government and society at large. The study utilizes survey data collected in Seoul in 2022.

## Methods

**Ethics statement:** The requirement to obtain informed consent was exempted by the Seoul Women and Family Foundation because there was no sensitive information, and the survey data were made available through the Korea Social Science Data Archive (KOSSDA) for public research, with all responses coded and anonymized.

### Study design

This study utilized a quantitative cross-sectional design, using survey data gathered in Seoul to investigate the FFI rate and its associated factors among Korean women of childbearing age. The research adhered to the STROBE guidelines for reporting quantitative cross-sectional studies (https://www.strobe-statement.org/).

### Study sample

The Survey on Low Birth Policy Demand in Seoul, conducted in 2022 by the Seoul Women and Family Foundation, targeted men and women aged 20 to 40 years. This survey aimed to gather insights into their perceptions of marriage and childbearing amidst South Korea’s declining birthrate. It encompassed a range of questions covering sociodemographic factors, perspectives on marriage and childbearing, employment status, income, sexual education, and gender inequality. The Seoul Women and Family Foundation reviewed and approved the data, which was then made available for public research through the Korea Social Science Data Archive (KOSSDA). All responses were coded and anonymized to ensure privacy. Further information about the survey is available on the Survey Foundation’s website [19].

This study assessed the FFI rate among women of childbearing age (i.e., 20–40 years), analyzing their sociodemographic characteristics and their perspectives on marriage, childbearing, and gender inequality. Out of the 568 Korean women who participated in the survey, 496 aged 26–40 years provided complete responses and were included in the analysis, yielding a response rate of 87.3%.

### Variables

#### Future fertility intentions

In this study, the FFI variable was identified as the dependent variable. We assessed the FFI data using a single survey question: “Do you intend to have (more) children in the future?” This question provided five response options: (1) “I want to,” (2) “I want to, if possible,” (3) “I do not want to,” (4) “I do not want to at all,” and (5) “I have never thought about it.” For analysis purposes, we redefined these responses into a binary variable. The “yes” category encompassed responses 1 and 2, while the “no” category included the remaining responses.

#### Sociodemographic characteristics

We included the following sociodemographic factors: age; marital status (married or unmarried, including single mothers); education level (high school or lower, college or university, and higher); employment status (regular worker, non-regular worker, self-employed, unpaid family worker, and unreported, as classified by the survey organization); weekly working time (no more than 20 hours, 21–40 hours, 41–50 hours, and more than 50 hours); number of children; age at first sexual education (elementary school, middle school, high school, adulthood, and never received); and monthly income (no more than 2 million Korean won [KRW], 2.01–4 million KRW, 4.01–6 million KRW, and more than 6 million KRW).

#### Perspectives on marriage, childbearing, and gender inequality

For our research criteria, we considered several perspectives on marriage, childbearing, and gender inequality. These included questions such as whether it is possible to have children without getting married, whether one must have children after marriage, whether children strengthen the relationship between a couple, whether motherhood is considered sacred, whether abortion should be allowed without penalty or disadvantage, whether Korean family culture is equal for women, and whether Korean workplace culture is equal for women. Each question could be answered with a simple “Yes” or “No.”

### Data collection and statistical analysis

The data utilized in this study are numeric and were sourced from the KOSSDA website (ver. 1). It has been publicly available since February 21, 2024. The dataset includes a comprehensive questionnaire in SPSS format, with all personal information anonymized, rendering it appropriate for research purposes.

Bivariate analyses were conducted to explore participant characteristics and estimate the FFI rate. The two-sided Fisher exact test was utilized to calculate *p*-values, assessing differences in FFI rates across various sociodemographic factors or perspectives [20]. A *p*-value of less than .05 was considered statistically significant. To identify predictive factors associated with FFI, we employed a multivariate logistic regression model, using the “No” category of the FFI variable as the reference [21]. We obtained *p*-values for parameter estimates using the Wald test, and assessed model fit with the likelihood ratio chi-square (LRC) test [22]. We reported odds ratios (ORs) derived from exponentiated regression coefficients, along with their corresponding 95% confidence intervals (95% CIs). ORs were considered statistically significant if their 95% CIs did not include the value of 1. Additionally, we conducted a subgroup analysis to assess FFI among childless women of childbearing age. All analyses were performed using R software ver. 4.4.0 (R Foundation for Statistical Computing, Vienna, Austria).

## Results

### Sociodemographic characteristics and variations in the future fertility intentions rate

The study included 496 women with an average age of 34.2 years (range, 26–40 years), and the overall FFI rate was 25.6%. Participants aged 26 to 35 years made up 50.8% of the sample and had an FFI rate of 29.0%, which was slightly higher than the FFI rate of 22.1% observed among those aged 36 to 40 years. Unmarried women comprised 61.5% of the respondents and had a higher FFI rate of 27.9% compared to 22.0% for married women, but the difference was not significant. Furthermore, 82.3% held a college or university degree, with an FFI rate of 27.5%, higher than those with only a high school diploma or lower (18.9%) or a graduate degree (15.7%). Regarding employment status, 53.8% were regular workers, and 3.2% were unpaid family workers. The FFI rate was highest among the self-employed (33.3%) and regular workers (28.5%), while unpaid family workers had a lower rate of 12.5%. On average, participants worked 30.5 hours per week. The FFI rate was lowest (19.3%) among those working 20 hours or less and highest (31.5%) among those working 41 to 50 hours per week. In terms of childbearing, 68.4% of participants were childless, while 2.2% had 3 to 4 children. FFI rates decreased as the number of children increased, from 28.6% among childless women to 9.1% among those with 3 to 4 children. The majority of participants (87.2%) received sexual education in high school or earlier, while 5.4% had never received sexual education. FFI rates were highest (48.7%) among those who received sexual education in adulthood and lowest (14.8%) among those with no sexual education. The average monthly income among participants was 3.21 million KRW (approximately 2,538 US dollars), exceeding the national average for women in 2022 (2.68 million KRW) [23]. Although 23.8% earned no more than 2 million KRW (1,581 US dollars) and 12.7% earned more than 6 million KRW (4,739 US dollars), no significant differences in FFI rates were observed across monthly income levels (Table 1).

### Differences in the FFI rate based on perspectives on marriage, childbearing, and gender inequality

Table 2 presents differences in the FFI rate based on participants’ views on marriage, childbearing, and gender inequality. Among those surveyed, 52.4% believed that it is possible to have children without getting married, which corresponded to an FFI rate of 24.2%. A significant 76.6% felt that having children is not necessary after marriage, with an FFI rate of 20.0%. Conversely, 66.9% who believed that children could strengthen a couple’s relationship reported an FFI rate of 32.5%. Participants who did not consider motherhood sacred, making up 21.6% of the sample, had a notably lower FFI rate of 4.7%. Regarding abortion, 90.3% supported it without penalty, and this group had an FFI rate of 24.9%. In terms of gender inequality, 71.0% perceived Korean family culture as unequal for women, which was associated with an FFI rate of 23.3%. Similarly, 75.2% viewed the Korean workplace culture as unequal, with an FFI rate of 23.6%.

### Results of subgroup analysis

**Figure 1.**
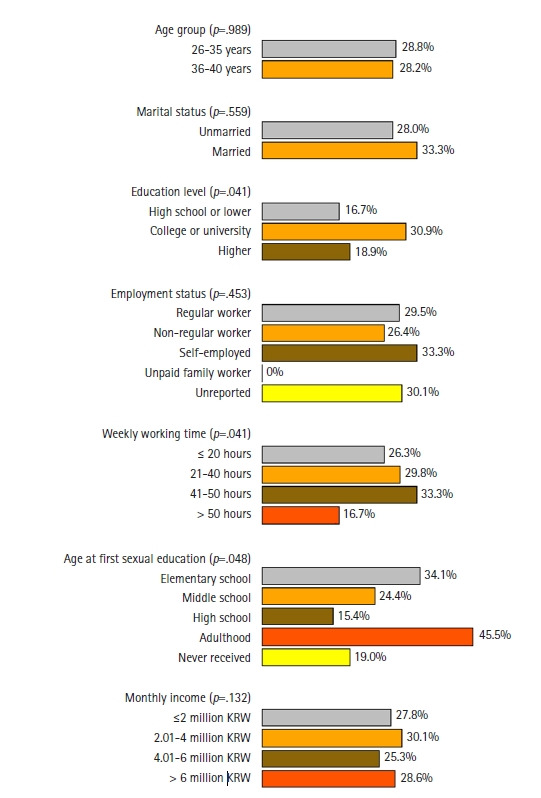
Differences in the future fertility intention (FFI) rate among childless women of childbearing age by sociodemographic characteristics. The two-sided Fisher exact test was utilized to calculate *p*-values, assessing differences in FFI rates across categories of each sociodemographic factor. KRW: Korean won (one million Korean won is approximately 790 US dollars).

**Figure 2.**
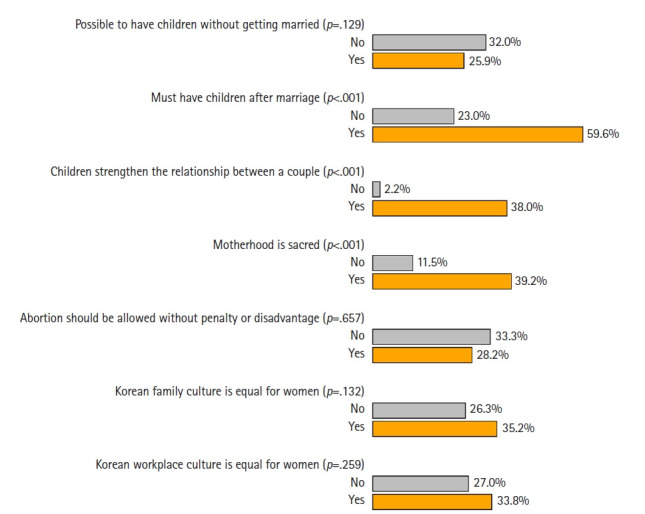
Differences in the future fertility intention (FFI) rate among childless women of childbearing age by perspectives towards marriage and childbearing. The two-sided Fisher exact test was utilized to calculate *p*-values, assessing differences in FFI rates across categories of each perspective.

Figures 1 and 2 illustrate the results of the subgroup analysis for childless women of childbearing age. Sociodemographic characteristics such as age, marital status, and employment status showed no significant differences in FFI rates, with the exception of unpaid family workers, who were absent from this subgroup. High FFI rates were observed in specific groups, including married women (33.3%), self-employed individuals (33.3%), those working 41 to 50 hours per week (33.3%), and those who received sexual education in elementary school (34.1%) or during adulthood (45.5%). Conversely, low FFI rates were found among those with a high school diploma or less (16.7%), those working more than 50 hours per week (16.7%), and those who received sexual education in high school (15.4%). Regarding views on marriage, childbearing, and gender inequality, high FFI rates were linked to beliefs that having children after marriage is necessary (59.6%), that children strengthen a couple’s relationship (38%), that motherhood is sacred (39.2%), and that there is gender equality in family culture (35.2%) and workplace culture (33.8%).

### Results of multivariate logistic regression analysis

Table 3 presents the factors associated with FFI in women of childbearing age, as determined by a multivariate logistic regression model. The model’s robustness and adequacy are evidenced by the low *p*-value obtained from the LRC test (<.00001).

The analysis identified several factors significantly associated with FFI, including education level, employment status, weekly working hours, number of children, age at first sexual education, and views on marriage, childbearing, and gender inequality (excluding the belief that having children outside of marriage is possible). Individuals working between 41 to 50 hours per week were found to have 2.49 times higher odds of experiencing FFI compared to those working 20 hours or less per week. Women who had never received sexual education exhibited significantly lower odds of FFI than those who were educated about it in elementary school (OR, 0.31; 95% CI, 0.17–0.54). In terms of perspectives, individuals who believed that having children after marriage is necessary, those who felt that children strengthen a couple’s relationship, and those who viewed motherhood as sacred were more likely to experience FFI, with odds 3.92, 6.45, and 2.24 times higher, respectively, compared to those with contrary views.

## Discussion

Our study examined factors affecting FFI among Korean women of childbearing age, taking into account sociodemographic characteristics and attitudes toward marriage and childbearing. We found that the overall FFI rate among these women was 25.6%, significantly lower than the 69.9% reported in a recent study on FFI [17]. This discrepancy may arise because the abovementioned study focused exclusively on newlyweds [17], who generally have a stronger desire for childbearing. In contrast, our study included both unmarried women (61.5%) and women with children (31.7%), which likely contributed to the lower FFI rate observed. Additionally, our data collection was confined to Seoul, a densely populated and costly city. The higher financial demands and increased pressures related to income, work, and lifestyle in Seoul might have further diminished the desire for children among women there, compared to those in other regions. This could help explain the discrepancies in findings.

Interestingly, we found that the FFI rate was highest among individuals with a college or university degree (27.5%) and lowest among those with a graduate degree (15.7%). A recent study highlighted educational disparities in the transition to first birth in South Korea [23], revealing that women with higher education tended to have fewer children than those with lower education. Additionally, women with a college or university degree were more likely to transition to first birth earlier than those with a graduate degree [24]. Our study further confirms this pattern, demonstrating that the desire to have children in the future was significantly higher among those with a college or university degree than among those with a graduate degree. A recent study found that higher levels of education significantly improved women’s employment prospects and earning potential, and the increased opportunity costs of childrearing may prompt women to delay or even reconsider having children [25]. Moreover, highly educated women often prioritize personal aspirations and self-fulfillment, placing greater emphasis on career advancement and self-actualization over childbearing [25].

We also observed that women who were either regular workers or self-employed exhibited high FFI rates. With the rising costs of childrearing and care, particularly in Seoul, regular workers and self-employed individuals—who typically have stable jobs and incomes—seem less anxious and may be more willing to have children than non-regular or unpaid family workers. However, these findings are based on a limited sample, in which some participants (24.6%) did not report their employment status. Future research exploring childbearing aspirations by employment status would benefit from a larger, more comprehensive dataset.

Our findings indicate that the age at which women first receive sexual education significantly influences FFI rates. The FFI rate was notably higher among those who received sexual education either in elementary school or during adulthood, and significantly lower among those who reported never having received it. Receiving sexual education early, in elementary school, may help girls understand gender differences and equip them to handle challenges such as sexual abuse or harassment, especially as early puberty becomes increasingly prevalent in modern society [26]. This early education may also enhance their appreciation for motherhood and foster a sense of societal responsibility, potentially encouraging women to have children.

Regarding perspectives on marriage and childbearing, we observed higher FFI rates among individuals who believed in having children after marriage (44.0%), those who thought that children strengthen relationships (32.5%), and those who considered motherhood sacred (31.4%). These findings are noteworthy because previous studies have not explored the impact of these perspectives on FFI. This highlights the importance for policymakers to focus on social welfare and enhance the rights of married women in childbirth and childcare, especially in light of South Korea’s critically low birth rate [6].

Our research shows that 76.6% of women of childbearing age do not consider having children after marriage to be mandatory, and 90.3% support the legalization of abortion, despite 78.4% viewing motherhood as sacred and 66.9% believing that having children strengthens the couple’s relationship. A recent social survey revealed that approximately 73% of Korean women feel that marriage and childbearing place them at a disadvantage [27]. They often face expectations to sacrifice significant aspects of their lives, such as careers and social connections, for housework and childcare—expectations that are seen as fair by men but unfair by women [8]. Furthermore, about 70.5% of women report experiencing discriminatory behavior both at home and in the workplace [27]. In this study, over 70% of participants acknowledged gender inequality in Korean family and workplace culture. However, these findings are based on a small sample of women of childbearing age and may not reflect the views of all Korean women. Future research with larger sample sizes is recommended.

This study also found that gender inequality in family and workplace culture significantly affected women’s FFI, as the FFI rate was markedly higher among those who perceived gender equality (31.7%). Despite many positive social changes, patriarchal family structures remain prevalent in South Korea, largely due to the enduring influence of Confucian ideals across East Asia [28]. Persistent stereotypes about gender roles within families, combined with a workplace culture that discourages higher birth rates, have placed significant pressure on women, who typically shoulder the majority of childcare responsibilities [29]. Social statistics reveal that, on average, women dedicate seven times more hours to housework and childrearing after marriage than men, while men spend 1.3 times more hours on paid work than women [29,30]. These significant disparities may contribute to young women’s increasing anxiety about marriage and their diminishing interest in starting families and having children.

Ultimately, it is crucial to recognize that promoting FFI and increasing the birth rate are responsibilities shared by society, and women should never be seen as mere instruments for forced childbirth. This study identified key factors influencing FFI among Korean women of childbearing age, highlighting the importance of addressing women’s mental health, implementing practical social welfare policies, providing comprehensive sex education, empowering women, and promoting gender equality to improve both FFI and birth rates.

This study has several limitations. First, it is a secondary analysis based on cross-sectional data and does not include a trend analysis for FFI. Second, the results may not be nationally representative, as the data were limited to Seoul. Furthermore, the generalizability of the findings might be compromised by the small sample size, and there may have been reluctance among some participants to provide sociodemographic information and personal perspectives on marriage and childbearing. Third, the study did not consider other potentially influential factors, such as health-related issues (health status, chronic conditions, disability, smoking, alcohol consumption, and physical activity) and social activities, because these were not included in the survey data. Future studies should aim to address these gaps as soon as relevant data become available.

This study evaluated the FFI rate among Korean women of childbearing age and identified influencing factors, utilizing data from Seoul. The analysis considered sociodemographic characteristics along with attitudes toward marriage, childbearing, and gender inequality. Elevated FFI rates were noted among the self-employed and individuals who received sexual education during adulthood. Higher rates were also observed in those who support the idea of having children post-marriage, believe that children solidify relationships, and perceive gender equality in their family or workplace environments. These insights are valuable for policymakers in Korea who are focused on enhancing birth rates through initiatives that emphasize social welfare, comprehensive sexual education, women’s empowerment, and gender equality. Additionally, nurses can leverage these findings to actively educate and provide healthcare that supports the physical and mental well-being of women, thereby improving their FFI.

## Figures and Tables

**Table 1.** Sociodemographic characteristics of participants and differences in the FFI rate (N=496)

Factor	Categories	n (%) or mean±SD	FFI rate (%)	*p* ^†^
All participants		496 (100)	25.6	
Age (year)		34.2±5.4		.118
	26–35	252 (50.8)	29.0	
	36–40	244 (49.2)	22.1	
Marital status	Unmarried^‡^	305 (61.5)	27.9	.147
	Married	191 (38.5)	22.0	
Education level	High school or lower	37 (7.4)	18.9	.031
	College or university	408 (82.3)	27.5	
	Higher	51 (10.3)	15.7	
Employment status	Regular worker	267 (53.8)	28.5	.032
	Non-regular worker	70 (14.1)	22.9	
	Self-employed	21 (4.2)	33.3	
	Unpaid family worker	16 (3.2)	12.5	
	Unreported	122 (24.6)	21.3	
Weekly working time (hour)		30.5±18.4		.038
	≤20	161 (32.5)	19.3	
	21–40	208 (41.9)	28.4	
	41–50	92 (18.5)	31.5	
	>50	35 (7.1)	22.9	
Number of children		0.5±0.8		.021
	0	339 (68.4)	28.6	
	1	77 (15.5)	23.4	
	2	69 (13.9)	15.9	
	3–4	11 (2.2)	9.1	
Age at first sexual education	Elementary school	181 (36.5)	29.8	.013
	Middle school	200 (40.2)	21.5	
	High school	51 (10.4)	15.7	
	Adulthood	37 (7.5)	48.7	
	Never received	27 (5.4)	14.8	
Monthly income (Korean won)^§^		3.2±2.4		.088
	≤2 million	118 (23.8)	19.6	
	2.01–4 million	237 (47.8)	27.9	
	4.01–6 million	78 (15.7)	24.8	
	>6 million	63 (12.7)	21.5	

FFI: future fertility intention.

† The two-sided Fisher exact test was used to calculate *p*-values for differences in the rate of FFI between categories of each sociodemographic factor.

‡ Including single mothers.

§ One million Korean won is approximately 790 US dollars.

**Table 2.** Differences in the FFI rate according to perspectives on marriage and childbearing (N=496)

Factor	Categories	n (%)	FFI rate (%)	*p* ^†^
Possible to have children without getting married	No	236 (47.6)	27.1	.272
	Yes	260 (52.4)	24.2	
Must have children after marriage	No	380 (76.6)	20.0	<.001
	Yes	116 (23.4)	44.0	
Children strengthen the relationship between a couple	No	164 (33.1)	11.6	<.001
	Yes	332 (66.9)	32.5	
Motherhood is sacred	No	107 (21.6)	4.7	<.001
	Yes	389 (78.4)	31.4	
Abortion should be allowed without penalty or disadvantage	No	48 (9.7)	31.3	.042
	Yes	448 (90.3)	24.9	
Korean family culture is equal for women	No	352 (71.0)	23.3	.038
	Yes	144 (29.0)	31.7	
Korean workplace culture is equal for women	No	373 (75.2)	23.6	.041
	Yes	123 (24.8)	31.7	

FFI: future fertility intentions.

† The two-sided Fisher exact test was used to calculate *p*-values for differences in the rate of FFI between categories of each perspective.

**Table 3.** Results of multivariate logistic regression analysis of the FFI rate (N=496)

Factors	Category	OR (95% CI)	*p*
Age group (year)	26–35	1	
	36–40	0.58 (0.25–1.05)	.082
Marital status	Unmarried^†^	1	
	Married	0.81 (0.61–1.17)	.413
Education level	High school or lower	1	
	College or university	1.46 (1.08–2.64)	.014
	Higher	0.84 (0.50–1.55)	.183
Employment status	Regular worker	1	
	Non-regular worker	0.61 (0.47–0.93)	.038
	Self-employed	1.23 (0.91–1.87)	.066
	Unpaid family worker	0.49 (0.21–0.83)	.038
	Unreported	0.58 (0.48–0.88)	.032
Weekly working time (hour)	≤20	1	
	21–40	2.37 (1.32–4.23)	.004
	41–50	2.49 (1.34–4.62)	.004
	>50	1.67 (0.84–3.30)	.139
Number of children	0	1	
	1	0.25 (0.15–0.39)	<.001
	2	0.11 (0.06–0.18)	<.001
	3–4	0.04 (0.02–0.14)	<.001
Age at first sexual education	Elementary school	1	
	Middle school	0.70 (0.25–1.29)	.064
	High school	0.54 (0.35–0.83)	.006
	Adulthood	2.05 (1.36–3.06)	<0.001
	Never received	0.31 (0.17–0.54)	<.001
Monthly income (Korean won)^‡^	≤2 million	1	
	2.01–4 million	1.43 (0.95–2.09)	.088
	4.01–6 million	1.21 (0.83–2.02)	.131
	>6 million	1.61 (0.92–4.42)	.072
Possible to have children without getting married	No	1	
	Yes	0.91 (0.73–1.13)	.419
Must have children after marriage	No	1	
	Yes	3.92 (3.01–5.11)	<.001
Children strengthen the relationship between a couple	No	1	
	Yes	6.45 (4.09–10.13)	<.001
Motherhood is a sacred	No	1	
	Yes	2.24 (1.69–2.96)	<.001
Abortion should be allowed without penalty or disadvantage	No	1	
	Yes	0.45 (0.31–0.65)	<.001
Korean family culture is equal for women	No	1	
	Yes	1.28 (1.05–1.68)	.022
Korean workplace culture is equal for women	No	1	
	Yes	1.36 (1.03–1.78)	.042

The odds ratio (OR) and corresponding 95% confidence interval (CI) were calculated against “no future fertility intentions” as the reference.

† Including single mothers.

‡ One million Korean won is approximately 790 US dollars.
